# Supplementary material for: Choosing between AR(1) and VAR(1) models in typical psychological applications
Source: PLoS One. 2020 Oct 29;15(10):e0240730. doi: 10.1371/journal.pone.0240730 (PMC7595444; doi:10.1371/journal.pone.0240730)
Supplement: S1 Fig — (PDF) [file pone.0240730.s001.pdf]

Sampling cells on the $D \times O$ grid S1 Fig shows the D and O values of the 10000 VAR models sampled from the mixed VAR model estimated from the "MindMaastricht" data [9]:

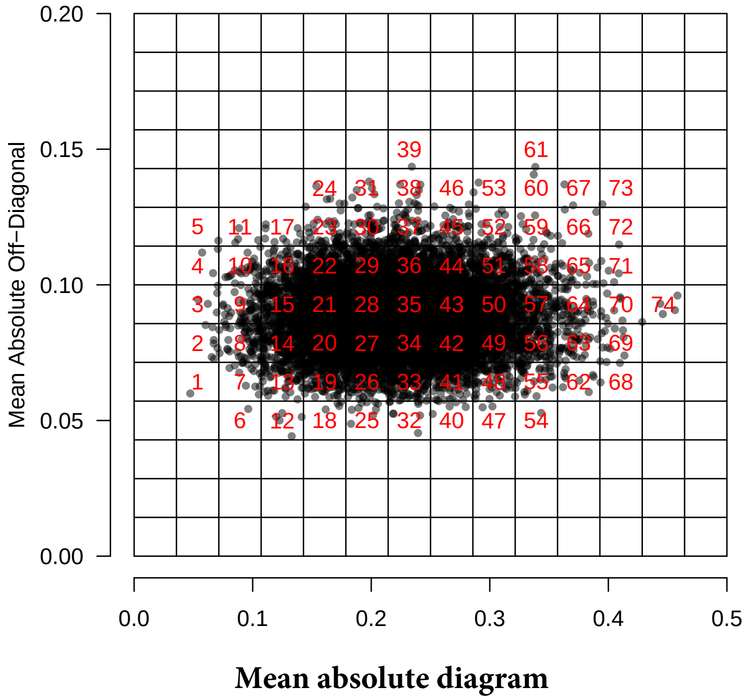

S1 Fig. D and O valiue for the initially sampled 1000 VAR models.

Each point in S1 Fig represents one of the 10000 VAR models we initially sampled from the mixed model. We see that there are 74 cells in which at least one model has been sampled. We then discarded these initial models and sampled from the mixed model until each of the 74 cells was filled with 100 models. We used these $74 \times 100 = 7400$ models in the simulation study reported in the main text.
